# Case of Mental Derangement, with Dissection

**Published:** 1831

**Authors:** 


### XLVII.

### Case of Mental Derangement, with Dissection.

Dr. Favell relates the case in the last number of the Phrenological Journal. On the 18th October, 1830, he visited Mrs. M. whom he had attended some months previously for an ulcer on the foot, and was struck with her altered appearance. She had now a vacant stare, dejected look, and evidently a mind ill at ease. She used only monosyllables — complained of no pain, and was averse to any exertion. Her pulse was 80, sleep regular, excretions natural. This state had existed for three or four months, without any obvious moral or physical cause. It was ascertained, however, that, for some five or six months past Mr. and Mrs. M. had not lived happily together. She had taken a dislike to some of Mr. M.'s children by a former wife, and whom she wished to be sent elsewhere. Dr. F. did not think that medicine would be of much use, and therefore only gave some directions respecting the diet, &c.

"It was on the 22d that I happened to call during dinner time, and I shall not soon forget her appearance on that occasion. She was set near the table, whilst her husband was carving a fowl, and as soon as he had taken off a leg, she immediately seized it, tore it to tatters with her fingers, and filled her mouth with the flesh, which she continued masticating for nearly a quarter of an hour. I staid with her for a considerable time, asked her various questions about herself, the fowl, her friends

&c. but all to no purpose, she would not allow a syllable to escape her. She had this day a large blister put down the nape of her neck, and reaching down betwixt her shoulders. On the 23d I saw her again, she was now very unwell and in bed,—her pulse had risen from 80 to upwards of 100,—her skin was hot, and the bowels had not been moved since the 21st. She would not allow me to see her tongue, and when I attempted a second time to feel her pulse, she made considerable resistance. I asked her if she had pain in her head, but I obtained no answer, and she continued to take no notice of whatever was done by the by-standers. Had I been ever so anxious to have had recourse to venesection, it would have been impossible to have performed the operation, from the manner in which she kept the fore-arm bent upon the humerus. She had a number of leeches applied to the temples and forehead. A purging draught was ordered to be given immediately, and a mixture with camphor and aq. ammon. acet. every three hours. I saw her the next day, the 24th. She had passed a restless night, her pulse was beating 130 strokes in a minute. The bowels had been freely moved, but they had not been able to give her more than one dose of the mixture. She would now answer me any question which I put to her, and complained of pain in the head. An additional number of leeches were applied to the temples, and cold cloths to the forehead. She died in the evening. On the following day, I obtained permission to open her head, which I did in the presence of my friends Dr. Holland and Mr. Gregory. There was nothing particular to be observed on the dura mater nor in the sinuses; but on the outer covering being removed, we perceived very evident traces of inflammatory action in the arachnoid membrane, which was opake and redder, with depositions of coagulable lymph. But the most interesting and important appearance was in the brain itself,—*the whole of the anterior lobe was in a state of disorganization*, presenting an unnatural greyish aspect; and the cerebral substance itself was so soft, that even the slightest touch was sufficient to destroy it. It is impossible to give an accurate description of it by words, but it has never fallen to my lot, and it had never fallen to the lot of either of my friends, to witness such extensive mischief. But it was most interesting to observe, that the *disease was entirely confined to the anterior lobes*; all the rest of the brain and cerebellum, from the fissure of Sylvius backward, being quite healthy, and as firm as any brain I ever dissected.

Now, how far do the appearances which we observed on dissection throw light upon the case? It appears to me clear, that the disease in the anterior lobes was not the consequence of any general inflammatory action in the brain, — else why was not the brain more extensively diseased? It was unconnected with the affection of the arachnoid, because the arachnoid presented similar appearances in every part of its extent; and the inflammatory affection of this membrane (which was the immediate cause of death) did not commence, I apprehend, till the 23d, whereas she had been labouring under disease for several months previously. Is it not most reasonable to consider that the unhappy differences which occurred in the family were the cause of the appearances which we observed in the anterior lobes of the brain; and that the effect of this was the state of mental alienation, in which she existed for the last several months of her life?"

---

